# A practical application of CP-ABE for mobile PHR system: a study on the user accountability

**DOI:** 10.1186/s40064-016-3002-y

**Published:** 2016-08-11

**Authors:** Hanshu Hong, Di Chen, Zhixin Sun

**Affiliations:** 1 Key Lab of Broadband Wireless Communication and Sensor Network Technology, Nanjing University of Posts and Telecommunications, Nanjing, China; 2 Inter-College Program in Genetics, Pennsylvania State University, University Park, PA USA

**Keywords:** CP-ABE, Mobile PHR system, User accountability

## Abstract

**Background:**

Attribute based encryption has been widely applied for secure data protection in PHR systems. However, since different users may share the same attributes in the system, a user may leaks his private key for illegal data sharing without being detected. This will add more threat to the private data stored in PHR system.

**Finding:**

To help users achieve higher efficiency and more secure data sharing in mobile PHR system, based on previous works, we study the traitor tracing mechanism in attribute based cryptosystem and propose a high efficient attribute based encryption with user accountability in mobile PHR system. If a malicious PHR user exposes his private key for illegal data sharing, his identity can be accurately pinpointed by the system manager. During the whole process of data sharing, no bilinear pairing operations are needed, hence this will the mobile terminal devices from heavy computation burden.

**Conclusion:**

As a further study, in this short report, we show that using a novel attribute based encryption with user accountability can help users achieve better efficiency and more secure data sharing in mobile PHR system.

## Background

Personal health record (PHR) (Zuckerman and Kim 2009; Koufi et al. 2014) contains massive private data in terms of the user’s health conditions, disease history, medication and other personal information. Due to the capability of improving the efficiency of healthcare, PHR has gained increasingly popularity nowadays and has been widely applied in the medical area such as diseases rehabilitation, disease prevention (McInnes and Shimada 2013), medical treatment, etc. Considering the private nature of PHR (Price et al. 2015), special encryption techniques should be implemented for protection in the PHR system (Liu et al. 2015; Sangeetha et al. 2014).

**Fig. 1:**
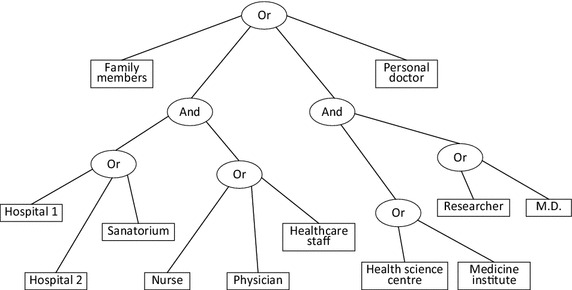
An CP-ABE access control structure for PHR data

Ciphertext policy attribute based encryption (CP-ABE) (Goyal et al. 2006; Waters 2011) was proposed by Waters in 2006 and has been considered suitable for access control for PHR system, since it reduces the encryption cost for PHR data owner and can also provide flexible self-centric data access management (Hong and Sun 2016; Fuji and Abbott 2012) at the same time. Unlike identity based cryptosystem (Li and Khan 2012), in CP-ABE, user’s access privileges are defined by a set of attributes. A user can read the ciphertext on condition that the attributes he owns match with the policy (Price et al. 2015; Hong et al. 2016). An illustration of ciphertext access policy is shown in Fig. 1, the PHR data owner may not know the exact identity of users who have the privileges to access the data, but can describe those using attributes such as “family members”, “Nurse”. For instance, if a user owns the attributes of {Hospital 1, Physician}, then he can get access to the PHR data since the attributes he possesses satisfy with the access structure illustrated in Fig. 1.

Many schemes have applied attribute based encryption to design medical care systems such as PHR (Qian et al. 2015; Liu et al. 2013; Li et al. 2015; Xhafa et al. 2015) and BAN (Tan et al. 2011; Tian et al. 2014), but the efficiency is still unsatisfactory. One important factor is that a PHR user has to run many times of bilinear pairing operations when decrypting a ciphertext. When PHR users get access to the encrypted data using mobile devices with restricted computing resources such as cellphones, body area sensors, smart watches, the heavy decryption computation will add difficulty in the process of mobile PHR data sharing.

Key abuse is another obstacle to apply attribute based encryption to PHR system. ABE is an advanced type of broadcast encryption, users owing the same attributes share the same private key. However at the same time, a malicious user may expose his private key deliberately without being detected. Thus, a mechanism which provides user accountability and traitor tracing should also be introduced.

Based on the previous works (Liu et al. 2013; Tan et al. 2011; Li et al. 2015; Tian et al. 2014; Xhafa et al. 2015; Li and Khan 2012; Hong and Sun 2016), to better solve the problems described above and help users achieve secure data sharing in mobile PHR system, the following constructions are established:

Firstly, we propose a user accountable ciphertext policy attribute based encryption without pairings (UA-CPABE-WP) for mobile PHR system. In our UA-CPABE-WP, users can recover the plaintext on condition that the possessing attributes satisfy with the access policy.

Secondly, the mechanism of user accountability is introduced. If a malicious PHR user exposes his private key for illegal data sharing, his identity can be accurately pinpointed by the system manager.

Thirdly, no bilinear pairing are needed during data sharing, hence relieving the mobile terminal devices from large calculation.

## Our studies

### Implementation of the proposed UA-CPABE-WP

**Fig. 2:**
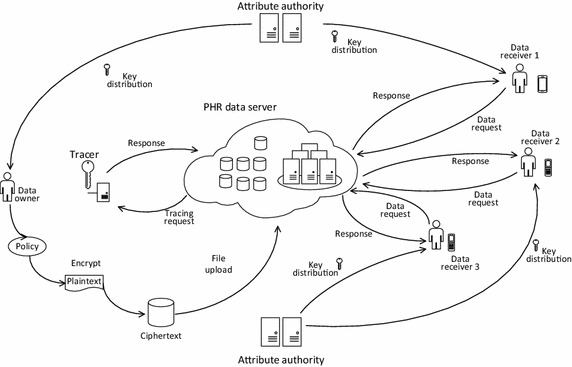
The implementation of our UA-CPABE-WP

The implementation example of our scheme can be illustrated in Fig. 2. It consists of 6 entitles: AA (Attribute authority), PHR data center, data owner and receiver. Base station and data center are hardware architectures which are responsible for mobile communications and file storage. AA generates attribute private key for each user in the system. PHR data center stores massive PHR data and responds to user’s data access request. Data owner and receiver are the two sides of communication, data owner encrypts the file with an access structure, while a receiver can decrypt the ciphertext using mobile devices if the attributes he owns match with the access structure. Tracer can pinpoint the exact identity of the traitor who leaks his private key deliberately.

### Constructions

Before introducing the formulized definitions of our scheme, some notations are defined in Table 1 for the convenience and clearness of description.

**Table 1:** Notations and their corresponding meanings

Notation	Meaning	Notation	Meaning
AA	Attribute authority	*PK*	Public parameters
*MK*	System master key	*A*_*i*_	A single attribute *i*
*MK*_*id*_	User’s private key	*id*	User’s identity
*CT*	Ciphertext	*M*	Plaintext

Our UA-CPABE-WP includes the following algorithms:

*Setup*: Let Let *G* to be a cyclic addition group with generator *q* and prime order *p*. Defines a global attribute set $\left\{ {A_{i} } \right\}$ and picks $t_{i} \in Z_{q}^{*}$ for each attribute in $\left\{ {A_{i} } \right\}$. Let $T_{i} = t_{i} p$. Picks secret numbers $h ,y \in Z_{q}^{*}$ and calculates $Y = yp,\;H = hp$. Define a hash function $H_{1} : \left\{ {0,1} \right\}^{ *} \to \left\{ {0,1} \right\}^{m} ,m$ is the size of plaintext.

The system public parameters are $\left\{ {G,q,p,A_{i} ,T_{i} , Y,H_{1} ,H} \right\}$ and the system master keys are $\left\{ {t_{i} ,y,h} \right\}$.

*Private key generation*: AA assigns a global unique identifier for each user in the PHR system. For a PHR user (without loss of generality, denote his identity by *id*) possessing attribute set *S*, AA generates his private key *SK*_*id*_ as follows:

1 $$SK_{id} :\left\{ {K = \left( {id \cdot y + r} \right)h^{ - 1} ,\forall A_{i} \in S,D_{i} = t_{i} - r} \right\}$$

*Encrypt*: When a data owner wants to share his private PHR data with some people processing certain attributes, he works as described below:

Picks a polynomial $q_{x}$ for each node $x$ for access control structure. Denote the degree of $q_{x}$ to be one less than the threshold value node. For the root node data owner sets $q_{root} \left( 0 \right) = s$. For any other node, let $q_{x} \left( 0 \right) = q_{parent\left( x \right)} \left( {index\left( x \right)} \right)$. The ciphertext is constructed as:

$$C_{0} = H_{1} \left( {sY} \right)M,\quad C_{1} = sH$$

2 $$C_{2,i} = q_{i} \left( 0 \right)p,\quad C_{3,i} = q_{i} \left( 0 \right)T_{i}$$

*Decrypt*: Upon receiving *CT*, data receiver calculates:

3 $$M = C_{0} \oplus H_{1} \left( {id^{ - 1} \left( {K \cdot C_{1} + \mathop \sum \limits_{i \in \gamma } \left( {D_{i} \cdot C_{2,i} - C_{3,i} } \right)} \right)} \right)$$

Correctness proof:

If $x$ is a leaf node,

4 $$\begin{aligned} \sum\limits_{{i \in \gamma }} {\left( {D_{i} \cdot C_{{2,i}} - C_{{3,i}} } \right)} & = \sum\limits_{{i \in \gamma }} {(t_{i} - r)} q_{i} \left( 0 \right)p - q_{i} \left( 0 \right)T_{i} \\ & = - rq_{i} \left( 0 \right)p \\ \end{aligned}$$

If $x$ is a non-leaf node,

Let $$i = index\left( z \right),S_{x}^{\prime } = \left\{ {index\left( z \right):z \in S_{x} } \right\}$$

$$F_{x} = \mathop \sum \limits_{{z \in S_{x} }} F_{z}^{{\Delta_{{i,S_{{x^{\prime } }} \left( 0 \right)}} }}$$

$$= \mathop \sum \limits_{{z \in S_{x} }} rp \cdot q_{z} \left( 0 \right)^{{\Delta_{{i,S_{{x^{\prime } }} \left( 0 \right)}} }}$$

$$= \sum\limits_{{z \in S_{x}}} {rp \cdot q_{{parent}} \left( z \right)^{{{(index(z))}} ^{{\Delta _{{i,S_{{x^{\prime } }} }} (0)}} }}$$

$$= \mathop \sum \limits_{{z \in S_{x} }} rp \cdot q_{z} \left( x \right)^{{\Delta_{{i,S_{{x^{\prime } }} \left( 0 \right)}} }}$$

5 $$= - q_{x} \left( 0 \right) \cdot rp$$

Then, the algorithm calculates the $F_{root} = - q_{root} \left( 0 \right) \cdot rp = - rsp$ by recursive function and computes:

$$M = C_{0} H_{1} \left( {id^{ - 1} \left( {K \cdot C_{1} + \mathop \sum \limits_{i \in \gamma } \left( {D_{i} \cdot C_{2,i} - C_{3,i} } \right)} \right)} \right)$$

$$= C_{0} H_{1} \left( {id^{ - 1} \left( {\left( {id \cdot y + r} \right)h^{ - 1} \cdot sH - rsp} \right)} \right)$$

$$= C_{0} H_{1} \left( {id^{ - 1} \left( {\left( {id \cdot y + r} \right)sP - rsp} \right)} \right)$$

$$= H_{1} \left( {sY} \right)MH_{1} \left( {syp} \right)$$

6 $$= M$$

## Results and discussion

### Security proof

#### **Theorem**

*UA-CPABE-WP is secure under chosen message attack if CDH assumption holds*.

#### *Proof*

If there exists an *Adversary* can break our UA-CPABE-WP with an advantage $\left( {t,\varepsilon } \right)$, then there exists a *Simulator* breaking the CDH assumption with an advantage of $\left( {t^{\prime } ,\varepsilon^{\prime } } \right)$ which satisfies:

$$t^{\prime } \le t + \left( {nq_{p} + 4n + 9} \right) \cdot t_{sm} + \left( { nq_{k} + 2n + 2} \right) \cdot t_{a}$$

7 $$\varepsilon^{\prime } \ge \frac{\varepsilon }{{e\left( {q_{k} + 1} \right)}} \cdot \left( {1 - \frac{1}{{2^{l} }}} \right)$$

In lemma (7), $q_{p}$ is the amount of public key queries in the challenge game.

The detail proof follows from that in (Liu et al. 2013).

### PHR user accountability

When a malicious user (denote *mid* as his unique identity and $SK_{mid}$ as the private key he owns) leaks his private key deliberately in the PHR system for illegal data sharing, then his identity can be exactly pinpointed by tracer. Two main methods can be adopted for traitor tracing as follows:

- Since user’ds private key is unique, if the amount of users is not huge, tracer can build a list recoding each private key with its corresponding user’s identity as Table 2 shows. When private key exposure happens, tracer searches the identifier which corresponds to the leaked private key in the list and the traitor is able to be exactly traced.

**Table 2:** List of each private key with its corresponding user’s identity

User’s identity	Corresponding private key
$id_{1}$	$SK_{{id_{1} }}$
$id_{2}$	$SK_{{id_{2} }}$
…	…
$id_{n}$	$SK_{{id_{n} }}$
- Upon receiving a legal private key $SK_{mid} = \left\{ {K = \left( {mid \cdot y + r} \right)h^{ - 1} ,\forall A_{i} \in S,D_{i} = t_{i} - r} \right\}$ from PHR system, tracer firstly recovers the attribute set belonging to the malicious user from $D_{i}$ and calculates $r$ as follows:
  8 $$r = D_{i} - t_{i}$$

Then, the identity can be pinpointed by:

9 $$mid = \left( {K \cdot h - r} \right) \cdot y^{ - 1}$$

### Efficiency evaluation

In this section, we will compare the efficiency of our scheme with other schemes which have also applied attribute based encryption to medical systems. In this report, the *Encrypt* algorithm will take $\left( {2n + 2} \right)$ times of multiplication operation, while the *Decrypt* algorithm will take $\left( {n + 2} \right)$ times of multiplication operation and $\left( {n + 1} \right)$ times of addition. Denote “Exp”, “Pair”, “Mul”, and “Add” to be exponential operation, pairing operation, multiplication and addition respectively. The detailed comparison results in terms of computation costs are shown in Table 3.

**Table 3:** Efficiency comparison

Scheme	Encrypt cost	Decrypt cost	User accountability
Li et al. (2015)	4n + 2 Exp	4n Pair	Yes
Tian et al. (2014)	(n + 3) Exp + 1 Pair	2n Pair + n Exp	No
Ours	(2n + 2) Mul	(n + 2) Mul + (n + 1)Add	Yes

Since the computation cost of bilinear pairing is much larger than that of multiplication and addition, it can be seen that the efficiency of our UA-CPABE-WP is higher since no bilinear pairings are needed.

## Conclusion

In this report we provide a high efficient data sharing method using attribute based encryption with user accountability (UA-CPABE-WP). In our scheme, data owner can achieve secure and self-centric access control over the PHR data. Besides, the mechanism of user accountability is introduced. If a malicious PHR user exposes his private key for illegal data sharing, his identity can be pinpointed exactly. The better efficiency and security makes UA-CPABE-WP to be a promising method for data protection in mobile PHR system.
